# *XRCC1*, but not *APE1* and h*OGG1* gene polymorphisms is a risk factor for pterygium

**Published:** 2010-06-04

**Authors:** Pei-Liang Chen, Kun-Tu Yeh, Yi-Yu Tsai, Hank Koeh, Yu-Ling Liu, Huei Lee, Ya-Wen Cheng

**Affiliations:** 1 Department of Pharmacy, Tungs’ Taichung MetroHarbor Hospital, Taichung, Taiwan; 2 Institute of Medicine, Chung Shan Medical University, Taichung, Taiwan; 3 Department of Pathology, Changhua Christian Hospital, Changhua, Taiwan; 4 Department of Ophthalmology, China Medical University Hospital, Taichung, Taiwan; 5 Department of Ophthalmology, Tungs’ Taichung MetroHarbor Hospital, Taichung, Taiwan; 6 Department of Ophthalmology, Changhua Christian Hospital, Changhua, Taiwan; 7 Institute of Medical Molecular Toxicology, Chung Shan Medical University, Taichung, Taiwan; 8 Division of Environmental Health and Occupational Medicine, National Health Research Institutes, Zhunan, Miaoli County, Taiwan, ROC; 9 Department of Medical Research, Chung Shan Medical University Hospital, Taichung, Taiwan

## Abstract

**Purpose:**

Epidemiological evidence suggests that UV irradiation plays an important role in pterygium pathogenesis. UV irradiation can produce a wide range of DNA damage. The base excision repair (BER) pathway is considered the most important pathway involved in the repair of radiation-induced DNA damage. Based on previous studies, single-nucleotide polymorphisms (SNPs) in 8-oxoguanine glycosylase-1 (*OGG1*), X-ray repair cross-complementing-1 (*XRCC1*), and AP-endonuclease-1 (*APE1*) genes in the BER pathway have been found to affect the individual sensitivity to radiation exposure and induction of DNA damage. Therefore, we hypothesize that the genetic polymorphisms of these repair genes increase the risk of pterygium.

**Methods:**

*XRCC1, APE1*, and h*OGG1* polymorphisms were studied using fluorescence-labeled Taq Man probes on 83 pterygial specimens and 206 normal controls.

**Results:**

There was a significant difference between the case and control groups in the *XRCC1* genotype (p=0.038) but not in h*OGG1* (p=0.383) and *APE1* (p=0.898). The odds ratio of the *XRCC1* A/G polymorphism was 2.592 (95% CI=1.225–5.484, p=0.013) and the G/G polymorphism was 1.212 (95% CI=0.914–1.607), compared to the A/A wild-type genotype. Moreover, individuals who carried at least one C-allele (A/G and G/G) had a 1.710 fold increased risk of developing pterygium compared to those who carried the A/A wild type genotype (OR=1.710; 95% CI: 1.015–2.882, p=0.044). The h*OGG1* and *APE1* polymorphisms did not have an increased odds ratio compared with the wild type.

**Conclusions:**

*XRCC1 (Arg399 Glu)* is correlated with pterygium and might become a potential marker for the prediction of pterygium susceptibility.

## Introduction

Pterygium is a chronic condition characterized by the encroachment of a fleshy triangle of conjunctival tissue into the cornea. The pathogenesis of pterygium is under investigation and several factors including ultraviolet radiation, immunoinflammatory process, virus infection, and genetic factors have been reported to be related to pterygial formation [1]. Epidemiological evidence suggests that UV irradiation plays an important role [1-3]. The noxious effects of UV irradiation are either directly by UV phototoxic effects or indirectly by formation of radical oxygen species (ROS) [4-6].

ROS are very harmful to cells because they injure cellular DNA, proteins, and lipids (called oxidative stress) [4-7]. Among the numerous types of oxidative DNA damage, 8-hydroxydeoxyguanosine (8-OHdG) has received considerable attention because of its demonstrated mutagenic potential and it is a ubiquitous marker of oxidative stress [7,8].

The base excision repair (BER) pathway is considered an important pathway involved in repair of radiation-induced DNA damage [9-11]. In particular, common single-nucleotide polymorphisms (SNPs) in the 8-oxoguanine glycosylase-1 (*OGG1*), X-ray repair cross-complementing-1 (*XRCC1*), and the apyrimidinic endonuclease-endonuclease-1 (*APE1*) genes in the BER pathway have been the most extensively studied for their influences in the individual sensitivity to radiation exposure and induction of DNA damage [12-18].

Polymorphisms in human 8-oxoguanine glycosylase 1 (h*OGG1*) may alter glycosylase function and an individual’s ability to repair damaged DNA, possibly resulting in genetic instability that can foster carcinogenesis. An amino acid change from serine to cysteine at codon 326 (Ser326Cys) is the most frequently studied SNP. Kohno et al. [19] observed a significantly lower capacity to repair 8-OHdG for the hOGG1-Cys326 protein than for the hOGG1-Ser326 protein.

Apurinic/apyrimidinic endonuclease/redox factor-1(APE1/Ref-1) is an essential enzyme in the BER pathway involved in the excision of abasic sites formed in DNA cleavage by OGG1. Several sequence variants were identified in *APE1*, including an amino acid change from aspartic acid to glutamic acid (Asp148Glu) in exon 5 that may be associated with hypersensitivity to ionizing radiation [12].

X-ray cross-complementing group 1 (XRCC1) is one of the major DNA repair proteins involved in the base excision repair pathway. A functional polymorphism in the *XRCC1* gene may lead to decreased DNA repair capacity and thus confer an inherited predisposition to cancer risk. Several variants of *XRCC1* have been described, including one affecting codon 194 in exon 6 that results in an arginine (Arg) to tryptophan (Trp) substitution and one affecting codon 399 in exon 10 that results in an arginine (Arg) to glutamine (Gln) change. Arg399Gln occurs in the vicinity of the Poly-ADP ribose polymerase (PARP) binding domain. The presence of the variant 399Gln has been shown to be associated with measurable reduced DNA repair capacity and increased risk of several types of cancers [12-14].

Recently, researchers have begun to use single nucleotide polymorphisms (SNPs) to identify the genes associated with pterygium [20-23]. Single nucleotide polymorphisms are the most abundant types of DNA sequence variation in the human genome, and the SNP marker has provided a good method for identification of complex gene-associated diseases and recognition of patients predisposing to the diseases [24,25].

Therefore, the aim of this study was to determine the relationship between *XRCC1* (Arg399Gln), h*OGG1* (Ser326Cys), and *APE1* (Asp148Glu) SNPs and pterygium.

## Methods

### Patients

Primary pterygial samples were harvested from 83 patients undergoing pterygium surgery at China Medical University Hospital and other institutions. Control blood samples were the hospital controls collected from patients without pterygium and pinguecula. This study was performed with the approval of the Human Study Committee at China Medical University Hospital.

### Genomic DNA of blood samples from pterygium patients and controls

Pterygium tissues from patients and venous blood samples from controls were obtained for the collection of genomic DNA. The blood cells were isolated by the Ficoll-Paque method. Frozen tissues were homogenized in 10 mM Tris, 0.1 M NaCl, 25 mM EDTA (pH 8.0), and 0.5% SDS on ice. The aqueous supernatant was incubated with RNase A and RNase T1 (250 mg/ ml; Sigma Chemical Co., St. Louis, MO) at 37 °C for 60 min, followed by proteinase K digestion (10 mg/ml; Merck, Darmstadt, Germany) at 55 °C for 12 h. The supernatant was extracted twice with phenol:chloroform: isoamyl alcohol (25:24:1, v/v/v). Then, sodium acetate (0.3 M final concentration) was added to the aqueous supernatant. DNA was precipitated with ethanol and dissolved in water.

### *XRCC1* (Arg399Gln), *OGG1* (Ser326Cys), and *APE1* (Asp148Glu) SNP analysis

The *XRCC1* Arg399Gln (rs25487), h*OGG1* Ser326Cys (rs1052133), and *APE1* Asp148Glu (rs3136820) polymorphisms were genotyped using TaqMan allelic discrimination assays (Applied Biosystems, Foster City, CA). Probes, primers and TaqMan universal PCR master mix were purchased from ABI. Briefly, the genomic DNA region containing one of the two SNPs was amplified separately using a PCR reaction. Each PCR reaction contained: 20.0 ng DNA, 12.5 μl TaqMan Universal PCR Master Mix, 1.25 μl 20× TaqMan SNP Genotyping Assay Mix (including sequence-specific forward and reverse primers and two TaqMan MGB probes: one probe labeled with VIC- dye detects the Allele 1 sequence, one probe labeled with FAM^TM^ dye detects the Allele 2 sequence), and 9.25 μl ultrapure water in a 25 μl reaction volume. Reactions were incubated at 95 °C for 10 min, then denatured at 92 °C for 30 s, annealed and extended at 60 °C for 1 min. The last two procedures went through the cycle 40 times. The final products were analyzed on an ABI StepOne system.

### Statistical analysis

Statistical analysis of frequency distributions was done by the χ^2^ test, and the correlations between various genotypes of *XRCC1*, h*OGG1*, and *APE1* of case and control groups were analyzed by statistical software SPSS 10.0 (SPSS, Chicago, IL). Adjusted odd ratios (ORs) and a 95% confidence interval (95% CI) on pterygium were evaluated for various factors using a multiple logistic regression model.

## Results

There were 50 males and 33 females in the pterygium group (age range from 50 to 83 years, mean of 57 years) and 126 males and 80 females in the control group (age range from 55 to 75 years, mean of 62 years). There were no significant differences between both groups in age and sex.

### Relationship of *XRCC1* but not *APE1* and h*OGG1* gene polymorphisms and pterygium

To verify the association of risk and the genetic change in the base excision repair (BER) pathway in pterygium development, the polymorphisms of *XRCC1, APE1*, and h*OGG1* in the pterygium and control groups were analyzed. The results of the genotypes of *XRCC1* (Arg399Gln), h*OGG1* (Ser326Cys), and *APE1* (Asp148Glu) in the pterygium and control groups are shown in Table 1. The analysis of the polymorphisms located at *XRCC1* codon 399 in pterygium showed that 31 (37.3%) were homozygous for the A/A genotype, 17 (20.5%) were homozygous for the G/G genotype, and 35 (42.2%) were heterozygous for the A/G genotype. There was a significant difference between the case and control groups in the *XRCC1* genotype (p=0.038). However, no clear patterns were observed between the pterygium and control groups for significant associations with the h*OGG1* and *APE1* polymorphisms.

**Table 1:** Genotype distribution of *XRCC1* (Arg399Gln), h*OGG1* (Ser326Cys) and *APE1* (Asp148Glu) genes among pterygium patients and control group.

**Gene**	**Pterygium group (n=83; %)**	**Control group (n=206; %)**	**p value**
***XRCC1***			
Arg/Arg	31 (37.3)	104 (50.5)	
Arg/Glu	35 (42.2)	80 (38.8)	
Glu/Glu	17 (20.5)	22 (10.7)	0.038
**h*OGG1***			
Ser/Ser	10 (12.0)	32 (15.5)	
Ser/Cys	37 (44.6)	102 (49.5)	
Cys/Cys	36 (43.4)	72 (35.0)	0.383
***APE 1***			
Asp/Asp	30 (36.2)	70 (34.0)	
Asp/Gln	37 (44.5)	98 (47.6)	
Gln/Gln	16 (19.3)	38 (18.4)	0.898

### The *XRCC1* polymorphism, but not the h*OGG1* and *APE1* polymorphism, is a risk factor for pterygium

To understand whether the genetic polymorphisms of *XRCC1* (Arg399Gln), h*OGG1* (Ser326Cys), and *APE1* (Asp148Glu) increased the risk of pterygium development, the different genotypes and the risk of pterygium were compared. The odds ratio of the *XRCC1* A/G polymorphism was 2.592 (95% CI=1.225–5.484, p=0.013) and the G/G polymorphism was 1.212 (95% CI=0.914–1.607), compared to the A/A wild-type genotype. Hence, individuals who carried at least one C-allele (A/G and G/G) had a 1.710 fold increased risk of developing pterygium compared to those who carried the A/A wild type genotype (OR=1.710; 95% CI: 1.015–2.882, p=0.044; Table 2). The h*OGG1* and *APE1* polymorphisms did not increase the odds ratio compared with the wild type (Table 2). The multiple logistic regression analysis showed that the *XRCC1* genotype is related to the risk of pterygium after adjusted with h*OGG1* and *APE1* polymorphisms. Subjects who were heterozygous or homozygous for the variant allele (399Glu) of *XRCC1* appeared to experience a higher risk of pterygium than those who were homozygous for the wild-type allele (399Arg) (OR: 1.758; 95% CI: 1.038–2.980, p=0.036; Table 3).

**Table 2:** Risk of pterygium in relation to SNPs in genes involved in oxidative DNA repair in a population-based sample.

**Gene**	**OR**	**95% CI**	**p value**
***XRCC1***			
Arg/Arg	1		
Arg/Glu	1.21	0.914–1.607	0.183
Glu/Glu	2.59	1.225–5.484	0.013
Arg/Glu or Glu/Glu	1.71	1.015–2.882	0.044
**h*OGG1***			
Ser/Ser	1		
Ser/Cys	1.077	0.721–1.610	0.716
Cys/Cys	1.600	0.708–3.615	0.258
Ser/Cys or Cys/Cys	1.343	0.627–2.873	0.448
***APE 1***			
Asp/Asp	1		
Asp/Gln	0.939	0.706–1.249	0.663
Gln/Gln	0.982	0.476–2.206	0.962
Asp/Gln or Gln/Gln	0.909	0.534–1.549	0.726

**Table 3:** The multiple logistic regression analysis of *XRCC1* (Arg399Gln), h*OGG1* (Ser326Cys),and *APE1* (Asp148Glu) genotypes and the risk of pterygium.

**Variable groups**	**Unfavorable/ favorable**	**OR (95% CI)**	**p value**
*XRCC1*	polymorphism /wild type	1.758 (1.038–2.980)	0.036
h*OGG1*	polymorphism /wild type	1.378 (0.640–2.966)	0.412
*APE1*	polymorphism /wild type	0.848 (0.493–1.458)	0.551

## Discussion

Theories on the pathogenesis of pterygium have implicated ultraviolet light exposure as a major causative factor. Evidence for sunlight exposure as one of the prime etiological factors has been derived from both case-control studies and prevalence surveys [26-28]. Gazzard et al. [29] indicated that pterygium was independently related to increasing age and outdoor activity. The noxious effects of UV irradiation are either directly by UV phototoxic effects or indirectly by formation of radical oxygen species (ROS) [4-6].

ROS is very harmful to cells, because they injure cellular DNA, proteins, and lipids (called oxidative stress) [4-7]. Among the numerous types of oxidative DNA damage, 8-hydroxydeoxyguanosine (8-OHdG) has received considerable attention because of its demonstrated mutagenic potential and it is a ubiquitous marker of oxidative stress [7,8]. Our unpublished data also indicated that the 8-OHdG DNA adducts in pterygium tissues were significantly higher than in the conjunctiva [30] (data not shown). Therefore, we suspect that the capability of the DNA repair enzymes in pterygium was reduced.

Common polymorphisms in DNA repair enzymes have been hypothesized to result in reduced capability to repair DNA damage [31,32]. Several reports have indicated that genetic factors play a role in the development of pterygium [33-39]. Besides, some races have a greater predisposition to pterygia; for example, Indians are affected more than Caucasians, Thais more than Chinese, and dark-skinned Africans more than pale-skinned Arabs [39]. Although genetic factors have been proposed to play a role in pterygium formation, there have only been a few studies to clarify this proposition and no specific gene was identified. To the best of our knowledge, this is the first study concerned with the role of the DNA base excision repair (BER) pathway in pterygium. Our study revealed that the *XRCC1* (Arg399Glu) polymorphism is associated with susceptibility to pterygium, but the h*OGG1* (Ser326Cys) and *APE1* (Asp148Glu) are not. This finding is not consistent with previous reports, which have shown that the h*OGG1* Ser326Cys polymorphism is associated with the risk of pterygium [20]. Kau et al. [20] indicated that the h*OGG1* Ser326Cys polymorphism is a risk factor for pterygium in Chinese people. The homozygous Cys/Cys genotype was more prevalent in pterygium patients than in the controls with the odds ratio being 2.2 [20]. In this study, no association between the h*OGG1* Ser326Cys polymorphism and pterygium risk could be due to sample size, gender distribution, and detection method which were different from a previous report [20]. In this previous report, 70 patients with pterygium and 86 healthy subjects were analyzed. The proportion of males in the two groups was 85.7 and 74.4%, respectively [20]. In our study, 83 patients with pterygium and 206 healthy subjects were studied and the proportion of males in the two groups was 56.8 and 61.1% which is different from the previous report [20]. In addition, we detected the *XRCC1, APE1*, and h*OGG1* polymorphisms using a SNP Shot assay kit. The sensitivity and specificity were different with PCR-RFLP [40]. Nevertheless, the effects of the h*OGG1* Ser329Cys polymorphism on pterygium risk in Taiwanese people necessitate an increase in the number of study populations for further investigations.

UV irradiation can produce a wide range of DNA damage and most DNA damage is repaired by the DNA repair system. Our previous report has indicated that the *Ku70* promoter T-991C polymorphism is correlated with pterygium [41]. In our present study, the polymorphism of X-ray repair cross complementary 1 (*XRCC1*), a major gene in the BER system, is associated with pterygium, but the polymorphisms h*OGG1* and *APE 1* are not associated. Therefore, we suggest different DNA repair systems may play different roles in pterygium. These repair systems could be the basis of future surveys. Further study on polymorphisms of the genes in other repair systems is necessary to clearly define the molecular mechanism of pterygium formation by UV irradiation.

In conclusion, *XRCC1* Arg399Glu is correlated with pterygium and might become a potential marker for the prediction of pterygium susceptibility. It also provides valuable insight into the pathogenesis of pterygium.
